# Endometriosis-associated massive ascites in a young Nigerian lady

**DOI:** 10.4314/gmj.v57i2.12

**Published:** 2023-06

**Authors:** Abiodun S. Adeniran, Munirdeen A. Ijaiya, Kazeem O.O. Ibrahim, Saidu Ibrahim, Olaleke O Folaranmi

**Affiliations:** 1 Department of Obstetrics & Gynaecology University of Ilorin, PMB 1515, Ilorin, Nigeria; 2 Department of Obstetrics & Gynaecology, University of Ilorin Teaching Hospital, PMB 1459, Ilorin, Nigeria; 3 Department of Pathology, University of Ilorin, PMB 1515, Ilorin, Nigeria; 4 Department of Pathology, University of Ilorin Teaching Hospital, PMB 1459, Ilorin, Nigeria

**Keywords:** Dysmenorrhea, endometriosis, haemorrhagic ascites, massive ascites

## Abstract

Endometriosis-associated massive haemorrhagic ascites is rare and poses a diagnostic challenge to the gynaecologist due to its resemblance to malignancies, especially ovarian malignancy. We report a 31-year-old nulligravida with progressive abdominal swelling, worsening dysmenorrhea, weight loss and a family history of ovarian tumour. Pelvic ultrasonography and Computed Tomography scans suggested an ovarian mass suspected to be an ovarian malignancy. Exploratory laparotomy revealed massive haemorrhagic ascites (8.6 litre) and multiple nodular masses on the anterior abdominal wall, omentum, bowel and pelvic organs, which were biopsied and confirmed on histopathology to be endometriosis. She had drainage of ascites and hormonal suppression using progestogen (Medroxyprogesterone acetate) with no recurrence in 15 months. Endometriosis should be considered in young, nulligravid women with dysmenorrhea, weight loss and ascites.

**Funding:**

None declared

## Introduction

Endometriosis is the presence of endometrial tissue (stroma and functional glands) outside the uterine cavity; it is found almost exclusively in women of reproductive age. It has been reported in 1.4-8.2% of women of reproductive age.^1^ The aetiology of endometriosis is unknown, but theories of its possible origin include retrograde menstruation leading to the implantation of viable endometrial cells into the peritoneal cavity, metaplasia of coelomic epithelium^2^ or the spread of endometrial progenitor (stem) cells to the peritoneum and after that to other sites.^3^ The presentations in endometriosis can be gynaecological (dyspareunia, low abdominal pain, dysmenorrhea or infertility) from pelvic endometriosis. At the same time, extra-pelvic manifestations include umbilical swelling, haematochezia, emphysema, cough, haemoptysis, chest pain or dyspnea.^4^ Very rarely, pelvic endometriosis has been reported to be associated with massive haemorrhagic ascites. Since it was first reported in 1954, endometriosis-associated ascites has been reported in 63 women aged 19 to 51 years; 63.0% were of African descent, 82.0% were nulliparous, and common presentations include abdominal distention, weight loss, dysmenorrhea and menometrorrhagia.^4^,^5^

Generally, endometriosis with ascites presents a diagnostic dilemma to the gynaecologist due to haemorrhagic ascites, which may mimic underlying malignancies, especially ovarian carcinoma. Other possible differentials include ectopic pregnancy, Meig syndrome, trauma, rupture of haemorrhagic ovarian cyst, ovarian cancer and similar conditions that present with ascites.^5^ Therefore, endometriosis is an uncommon differential of ascites, especially in the presence of abdominal distention, weight loss, and a family history of ovarian pathology in the case reported. This case report aims to report another of these uncommon conditions to increase the index of suspicion among medical practitioners and add to the few cases reported globally.

## Case Report

A 31-year-old single, Para 0^+0^ lady presented at the gynaecology clinic with a history of progressive abdominal swelling of four months duration. There was associated worsening dysmenorrhea and menorrhagia, early satiety, weight loss and abdominal pain but no change in bowel habit.

Her elder sister had an exploratory laparotomy for an ovarian tumour five years prior to the presentation; the patient was unaware of the final diagnosis or other treatments offered, but the relative was alive and healthy. Menarche was at 11 years, and she menstruates for five days in a regular 28-day cycle; she was yet to initiate sexual activity.

On examination, there was no pallor, jaundice or peripheral lymphadenopathy; cardiovascular and respiratory systems evaluations were normal. The abdomen was grossly distended with ascites. Pelvic examination was deferred, and digital rectal examination was normal. Investigations showed normal results for full blood count, serum electrolyte, urea and creatinine, urinalysis, clotting profile, liver function tests, viral markers and electrocardiogram, while chest radiograph showed no pleural effusion. An abdominopelvic ultrasound scan revealed massive ascites, a bulky uterus with multiple uterine fibroid nodules and a right, uniformly cystic adnexal mass measuring 5.0 x 4.8cm. Abdominopelvic computerized tomography (CT) scan showed massive ascites and a right adnexium lobulated, solid complex mass measuring 5.0 x 4.3cm. All other intrabdominal organs were normal, and there was no mesenteric lymphadenopathy. The differential diagnosis was a malignant right ovarian tumour on a background of a uterine fibroid to rule out leiomyosarcoma.

The patient was counselled on the results and the benefit of exploratory laparotomy for staging and possible total abdominal hysterectomy with bilateral salpingo-oophorectomy. Exploratory laparotomy revealed massive haemorrhagic ascites (8.6 litre) with extensive nodular masses on the anterior abdominal wall, omentum, bowel and pelvic organs measuring about 2-4cm in diameter; the masses were adherent and bled on contact. There were pelvic adhesions involving the uterus, ovaries and fallopian tubes with multiple dark endometriotic cysts seen on the surfaces of the uterus and ovaries measuring 2-4cm in diameter. The uterus was bulky containing multiple fibroid nodules (myomectomy was performed with the removal of five nodules). The ovaries were released following adhesiolysis. The right ovary was slightly enlarged but otherwise normal, while the left ovary was grossly normal; multiple (three) biopsies were taken from each ovary. The liver surface and undersurface of the diaphragm were free of any lesions. Samples sent for evaluation included ascitic fluid for cytology. In contrast, the myomectomy specimens and biopsies of the multiple nodular masses from the uterus and ovaries were sent for histopathology.

**Figure 1:**
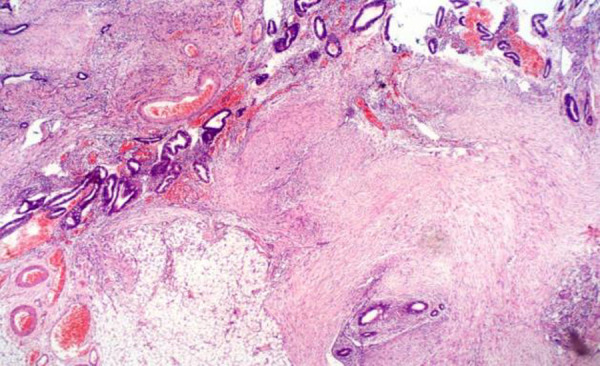
Histologic section showing omental tissue with endometrial tissue comprising glands and stroma embedded in adipose and fibrous tissue (H/E X 50)

**Figure 2:**
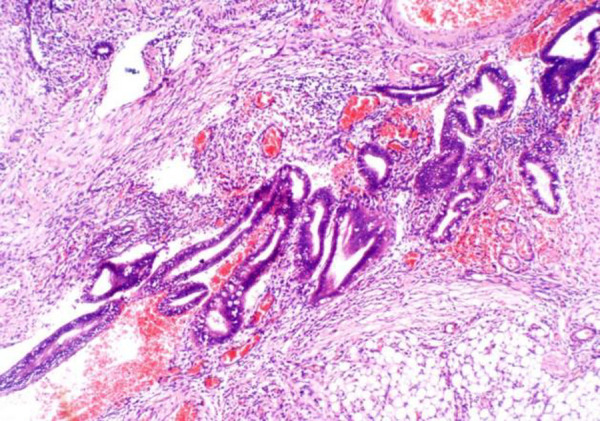
Histologic section showing a close-up of the endometrial tissue comprising straight and coiled glands composed of a single layer of columnar epithelium. The stroma comprises sheets of uniform round to oval cells with scanty cytoplasm. There are no features of atypia (H/E X 100)

The post-operative period was uneventful; she was discharged on the sixth post-operative day with a 3-week appointment to attend the gynaecology clinic. The follow-up visits have revealed satisfactory progress. The ascitic fluid cytology was negative for malignant cells. At the same time, the histopathology result showed multiple islands of endometrial tissue (glands and stroma) with no atypia, hyperplasia or malignancy and was suggestive of endometriosis (figures 1 & 2). The fibroid nodules were confirmed leiomyomata, while the ovarian tissue sample showed normal histology. The patient was counselled and commenced on intramuscular Medroxyprogesterone acetate 150mg every three months and is still on follow-up with no recurrence of the ascites.

## Discussion

We have presented a 31year old nulligravida with massive ascites, dysmenorrhea, weight loss and a family history of ovarian tumour who had exploratory laparotomy and biopsy with histopathology confirmation of extensive endometriosis and leiomyoma but no evidence of malignancy.

Massive ascites is a rare presentation of endometriosis; generally, a combination of massive ascites and weight loss suggests a malignancy, especially ovarian. When this combination is due to endometriosis, it presents a diagnostic dilemma to the gynaecologist resulting in a delay in diagnosis with adverse effects on the quality of life.^6^ Previous reports of a pre-operative diagnostic dilemma as a result of the similarity of endometriosis-associated ascites to ovarian malignancy included a woman who received pre-operative cytotoxic chemotherapy followed by exploratory laparotomy,^7^ misdiagnoses as carcinomatosis peritonei^8^ and mucinous ovarian neoplasia with pseudo-myxoma-peritonei.^9^ The family history of ovarian tumour heightened the suspicion of ovarian malignancy in the case presented in a first-degree relative.

Similar to the case presented, endometriosis-associated ascites has been reported to be commoner among women of African descent^4^,^10^, although it has been reported among Hispanic^11^ and Asian^8^,^9^ women. Endometriosis-associated ascites may occasionally present with coexisting pleural effusion^11^, although this was absent in the case presented. Also, affected women may be with^10^,^11^ or without^7^,^8^ preceding diagnosis of endometriosis; the patient presented belonged to the latter group. The aetiology of ascites in endometriosis remains elusive. Although Bernstein et al. 12 suggested irritation of serosal surfaces from the release of blood and endometrial cells following the rupture of endometrioma, it has been reported in cases without rupture of an endometrioma.^10^

In a review of 18 previous case reports of endometriosis-associated ascites, affected women were generally young (mean age of 27 years; age range19 to 47 years) nulliparous women. The commonest symptoms were increased abdominal girth, abdominal pain and dysmenorrhea. The ascitic fluid ranged from 150 to 7500ml, while the ascitic fluid was negative for malignant cells on cytology^11^, similar to the case presented. The non-specificity of the results hinders pre-operative evaluation. While the Cancer antigen (CA-125) level is generally elevated, abdominopelvic ultrasound may show ascites with^8^,^9^ or without adnexal mass, while Computerised Tomography (CT) scan may show homogenous, ill-defined enhancing cystic masses.^7^,^9^

The management includes exploratory laparotomy to drain the ascites to relieve symptoms and enable a biopsy to confirm the diagnosis. Additional medical or surgical treatment depends on several factors, including patient preference, disease severity and desire for future fertility.^6^ Medical management with hormones (progestin, combined progestin and estrogen, danazol, GnRH agonist, Ulipristal acetate) can cause suppression of ovarian function and cessation of menses^11^ while relieving symptoms. Surgical treatment may be conservative (removing or ablating visible endometriotic deposits) or radical (total hysterectomy and bilateral salpingo-ophorectomy).^6^-^11^ Miss. FO had laparotomy with drainage of the haemorrhagic ascites and excision of endometriotic nodules because she desires future fertility; she had been on follow-up for 15 months before this article was written.

Endometriosis-associated ascites may recur,^11^ This necessitates long-term follow-up and therapy. However, in the presence of associated infertility, this poses a management challenge because most treatment modalities cause a pseudo-menopausal state that does not favour fertility. Benaglia et al. 13 reported that controlled-ovarian hyperstimulation and consequent in-vitro fertilisation were not associated with a higher endometriosis recurrence rate.

### Patient consent

The patient gave informed consent to publish this case report with the assurance that no information that could identify her would be included.

## Conclusion

Endometriosis with haemorrhagic ascites is a rare condition that may be misdiagnosed as ovarian malignancy. A high index of suspicion should be entertained when evaluating young reproductive-age women with ascites and dysmenorrhea with/without ovarian enlargement. Gynaecologists should remember endometriosis as a differential in these women to reduce the negative impact on the patient's quality of life and facilitate early diagnosis.
